# Trefoil Factor Family (TFF) Modules Are Characteristic Constituents of Separate Mucin Complexes in the *Xenopus laevis* Integumentary Mucus: In Vitro Binding Studies with FIM-A.1

**DOI:** 10.3390/ijms21072400

**Published:** 2020-03-31

**Authors:** René Stürmer, Jana Reising, Werner Hoffmann

**Affiliations:** Institute of Molecular Biology and Medicinal Chemistry, Otto-von-Guericke University Magdeburg, Leipziger Str. 44, 39120 Magdeburg, Germany

**Keywords:** frog skin, mucin, lectin, mucous gland, TFF, trefoil factor, frog integumentary mucin, FIM-A.1, mucous gland self-renewal

## Abstract

The skin of the frog *Xenopus laeevis* is protected from microbial infections by a mucus barrier that contains frog integumentary mucins (FIM)-A.1, FIM-B.1, and FIM-C.1. These gel-forming mucins are synthesized in mucous glands consisting of ordinary mucous cells and one or more cone cells at the gland base. FIM-A.1 and FIM-C.1 are unique because their cysteine-rich domains belong to the trefoil factor family (TFF). Furthermore, FIM-A.1 is unusually short (about 400 amino acid residues). In contrast, FIM-B.1 contains cysteine-rich von Willebrand D (vWD) domains. Here, we separate skin extracts by the use of size exclusion chromatography and analyze the distribution of FIM-A.1 and FIM-C.1. Two mucin complexes were detected, i.e., a high-molecular-mass Complex I, which contains FIM-C.1 and little FIM-A.1, whereas Complex II is of lower molecular mass and contains the bulk of FIM-A.1. We purified FIM-A.1 by a combination of size-exclusion chromatography (SEC) and anion-exchange chromatography and performed first in vitro binding studies with radioactively labeled FIM-A.1. Binding of ^125^I-labeled FIM-A.1 to the high-molecular-mass Complex I was observed. We hypothesize that the presence of FIM-A.1 in Complex I is likely due to lectin interactions, e.g., with FIM-C.1, creating a complex mucus network.

## 1. Introduction

For a long time, *Xenopus laevis* skin secretions have been extensively studied because they are a rich source for biologically active peptides, which are known for their hormone-like (such as caerulein [1]) and antimicrobial activities (such as PGLa/PYLa [2]). These peptides are synthesized in granular/serous glands [3], which are more frequent in the dorsal part of the skin. Granular glands are able to regenerate after depletion by cell proliferation [4], probably from a yet unknown set of stem and precursor cells.

As an aquatic animal, *X. laevis* protects its skin from infections not only by antimicrobial peptides but also with a mucus barrier, which prevents attachment of microbia and supports the clearance of microorganisms. For example, infection with the fungus *Batrachochytrium dendrobatididis* results in the lethal skin disease chytridiomycosis in amphibians [5]. Of note, knockdown of the skin mucin MucXS (formerly: Otogelin-like) in *X. tropicalis* tadpoles led to increased susceptibility to infection of these animals with *Aeromonas hydrophilia* [6]. The frog integumentary mucus is expected to be a complex mixture of a huge variety of proteins, including gel-forming mucins; the latter are typical secretory products of the skin mucous glands [7]. Generally, gel-forming mucins appeared early in metazoan evolution, and the number of genes increased markedly in *Xenopus* [8]. Of special note, the cystic fibrosis transmembrane conductance regulator (CFTR) is also expressed in *X. laevis* mucous glands, probably regulating both the chloride and the HCO_3_^−^ contents of the mucus [9]. These ions play a key role in the viscoelastic and adhesive properties of mucous gels [10,11].

**Figure 1:**
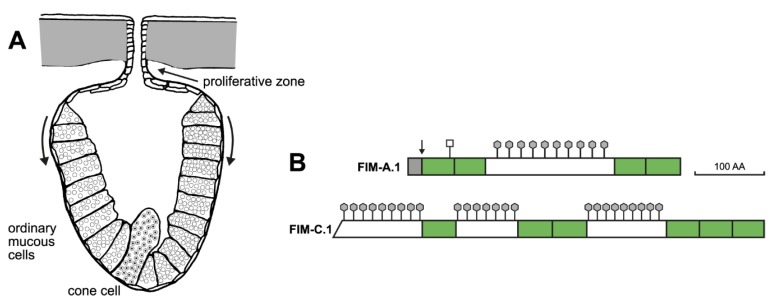
Schematic structure of a mucous gland from *X. laevis* skin (**A**) as well as the integumentary mucins FIM-A.1 and FIM-C.1 (**B**). (**A**) The postulated migration of ordinary mucous cells towards the base of the gland during self-renewal is indicated by arrows. Also shown are the different types of secretory granules in ordinary mucous and cone cells, respectively. (**B**) The TFF domains in FIMs are shown in green, highly O-glycosylated regions typical of mucins are indicated by hexagons, and a potential N-glycosylation site is indicated with a square. The arrow in FIM-A.1 represents the cleavage site in the precursor by signal peptidase.

The predominant mucin from *X. laevis* skin is frog integumentary mucin (FIM)-A.1 [12,13]. This is an unusual mucin because it is rather small (about 400 amino acid residues) and not related to the characteristic gel-forming mucins of mammals containing cysteine-rich von Willebrand D (vWD) domains [8,14]. The four cysteine-rich domains in FIM-A.1 rather belong to the trefoil factor family (TFF) domains (Figure 1B), which normally occur as secretory TFF peptides (formerly P-domain peptides) in manifold mucous epithelia from frog to human, e.g., mammalian TFF1 (one TFF domain), TFF2 (two TFF domains), and TFF3 (three TFF domains) [15,16,17,18,19]. In addition, polymorphic forms of FIM-C.1 have been partially characterized, where at least even five TFF domains were detected (Figure 1B) [20]. Furthermore, mucin FIM-B.1 was also discovered to contain typical vWD domains [21,22,23], such as *X. tropicalis* MucXS and mammalian MUC2, MUC5AC, MUC5B, MUC6, and MUC19 [6,8]. These secretory mucins are known to assemble to oligomers [24]. Taken together, the structure of the *X. laevis* integumentary mucus network appears to be structurally different and more complex than mammalian mucus. In the latter, generally, a combination of a predominant vWD-type mucin and a specific TFF peptide is synthesized from a specialized mucous cell, such as MUC6 and TFF2 in gastral gland cells (for compilation, see [18,25]).

The mucous glands of *X. laevis* skin consist of ordinary mucous cells and up to four cone cells at the base of these glands (Figure 1) [13]. From a morphological point of view, cone cells are quite different because they typically contain electron-dense core granules [26]. FIM-A.1 is localized within ordinary mucous, but not in cone cells [13]. In contrast, FIM-B.1 and FIM-C.1 appear predominantly in cone cells [26]. Of note, FIM-B.1 transcripts were detectable also at the base of ordinary mucous cells [21]. Thus, the localization of FIM-B.1 protein and transcripts does not seem to be congruent. This situation is reminiscent of TFF2 and MUC6 in human gastric fundic glands [27]. Here, TFF2 and MUC6 transcripts are localized in proliferating precursors of mucous neck cells, whereas the proteins are present in the mature cells underneath, which then further transdifferentiate into zymogenic cells during their migration towards the base of these glands [28]. In agreement with the observation of proliferating cells in the duct region of *X. laevis* mucous glands [26], it is tempting to speculate that these glands show continuous self-renewal by differentiation from stem and precursor cells followed by migration of cells towards the base of the glands where ordinary mucous cells finally transdifferentiate into cone cells (Figure 1A).

Currently, the function of TFF modules in FIMs has not been elucidated. Generally, TFF peptides are known for their lectin activities. For example, TFF1 and TFF3 bind to a lipopolysaccharide of *Helicobacter pylori* [29], whereas TFF2 and probably also the *X. laevis* ortholog xP4 bind to the evolutionary conserved GlcNAcα1→ 4Galβ1→ R moiety of the mucin MUC6 [30,31]. Furthermore, shuffled TFF modules are also present in other mosaic proteins, such as human zona pellucida proteins ZP1 and ZPB, and the sugar degrading enzymes sucrose-isomaltase, α-glucosidase, and maltase-glucoamylase [25]. Particularly, the occurrence in the latter is again indicative of the sugar-binding activity of TFF modules. Thus, it is within the limits of expectation that TFF modules in FIM-A.1 and FIM-C.1 interact with the highly O-glycosylated moieties of mucins and form a complex mucus network.

Here, we analyzed the mucus from *X. laevis* skin biochemically concerning FIM-A.1 and FIM-C.1, using size-exclusion chromatography (SEC). The aim is to check whether the unusual mucins containing TFF domains (i.e., the short FIM-A.1 and the much-longer FIM-C.1) form a tight complex. To accomplish this, we used separation on a Sephacryl S-500 HR (S-500) column because, here, even the human mucins MUC5AC and MUC6 appear as different entities [32]. We also purified the unusually short FIM-A.1 via a combination of SEC and anion-exchange chromatography, labeled FIM-A.1 radioactively with ^125^I, and performed first in vitro binding studies. This is a further step towards understanding the molecular function of TFF domains in mucins and to get a glimpse of the complexity of the frog integumentary mucus network.

## 2. Results

### 2.1. Characterization of X. laevis Skin Extracts by SEC and Western Blot Analysis

**Figure 2:**
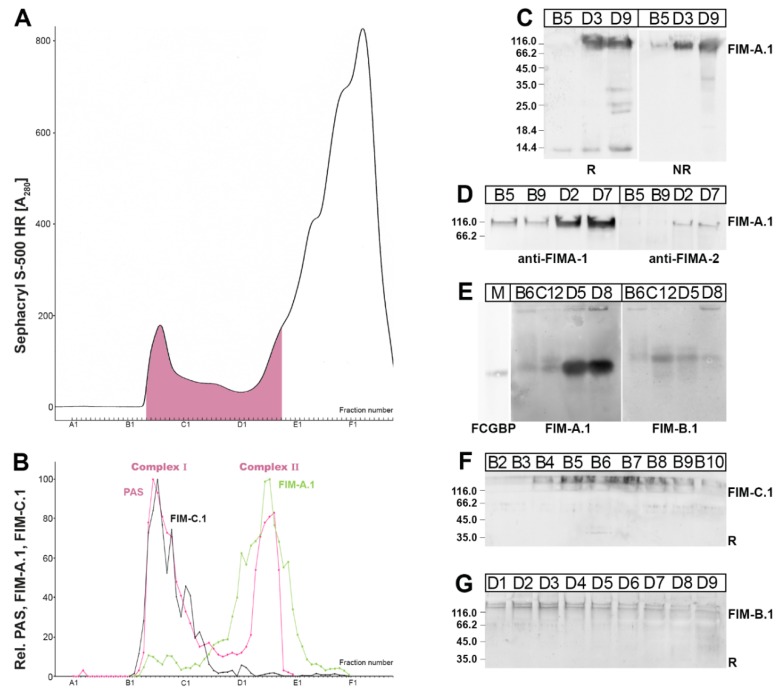
FPLC purification and analysis of FIM-A.1 and FIM-C.1 from a ventral *X. laevis* skin extract. (**A**) Elution profile after SEC on an S-500 column as determined by absorbance at 280 nm (PAS-positive mucin fractions: pink). (**B**) Distribution of the relative FIM-A.1 (green) and FIM-C.1 content (black) as determined by Western blot analysis under reducing conditions and semi-quantitative analysis of the characteristic ≥ 116,000 band (antiserum anti-FIMA-1) or the ≫ 116,000 smear (antiserum anti-FIMC-1). The mucin content was semi-quantitatively analyzed using the PAS reaction (pink; Complexes I and II are marked). (**C**) 15% SDS-PAGE under reducing (R) or non-reducing (NR) conditions and subsequent Western blot analysis of the fractions B5, D3, and B9 using anti-FIMA-1. Molecular mass standard: left. (**D**) 15% SDS-PAGE under reducing conditions of the fractions B5, B9, D2, and D7 and Western blot analysis using antiserum anti-FIMA-1 or anti-FIMA-2. (**E**) 1% AgGE of the fractions B6, C12, D5, and D8 and Western blot analysis using anti-FIMA-1 or anti-FIMB-1. Molecular mass standard (M): FCGBP from human colonic mucus. (**F**) 15% SDS-PAGE of fractions B2–B10 under reducing conditions and Western blot analysis using anti-FIMC-1. (**G**) 15% SDS-PAGE under reducing conditions of fractions D1–D9 and Western blot analysis using anti-FIMB-1.

*X. laevis* skin extracts were separated by SEC on an S-500 column (Figure 2). Periodic acid-Schiff (PAS) positive mucins were detectable within a relatively broad region with two maxima (Figure 2B), i.e., a high-molecular-mass Complex I (peak: B5–B8) also visible in the UV-absorption profile at 280 nm (Figure 2A), and Complex II with a lower molecular mass (peak: D4–D9). FIM-A.1 mainly appeared in Complex II, and only a minority was present in Complex I (Figure 2B). In contrast, FIM-C.1 was mainly associated with the high-molecular-mass Complex I (Figure 2B).

On denaturing SDS-PAGE, FIM-A.1 was detectable as a band with a relative molecular mass (Mr) of ≥116,000 under both reducing and non-reducing conditions (Figure 2C). Additionally, under reducing conditions, a band below 14,000 was also detected (Figure 2C). The ≥116,000 band was recognized with both the antiserum anti-FIMA-1 against the C-terminal as well as the N-terminal antiserum anti-FIMA-2 (Figure 2D). After non-denaturing agarose gel electrophoresis (AgGE; Figure 2E), FIM-A.1 in Complex II (fractions D5, D8) appeared as a very strong band with a Mr comparable with that of IgG Fc binding protein (FCGBP; Mr > 650,000, [33]). In contrast, FIM-A.1 in Complex I (fractions B6, C12) was only faintly visible and showed an additional diffuse smear with a higher Mr.

FIM-C.1 immunoreactivity was detectable in the high-molecular-mass range (Mr ≫ 116,000), peaking between fractions B4 and B9 (Figure 2F) and ending in about fraction C5. The Mr of FIM-C.1 is higher than that of FIM-A.1. Unfortunately, the anti-FIMC-1 antiserum did not react after AgGE, i.e., under non-denaturing conditions (data not shown).

For comparison, the distribution of FIM-B.1 was also analyzed (Figure 2G). A double band with a Mr ≫ 116,000 was visible with a broad distribution between fractions B5 and about D9. The anti-FIM-B.1 antiserum also recognized a band after AgGE with a Mr higher than that of FIM-A.1 (Figure 2E).

**Figure 3:**
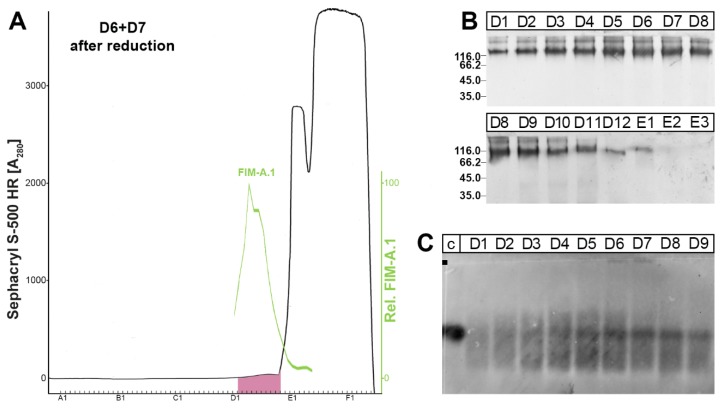
Analysis of *X. laevis* FIM-A.1 by SEC after reduction. Fractions D6 + D7 (1.5 mL each) from SEC of Figure 2 were reduced in boiling 4.7% β-mercaptoethanol/0.1% SDS and separated immediately thereafter by SEC on an S-500 column. (**A**) Elution profile as determined by absorbance at 280 nm (PAS-positive mucin fractions: pink). Relative FIM-A.1 content (green), as determined by Western blot analysis under reducing conditions and semi-quantitative analysis of the characteristic ≥ 116,000 band (anti-FIMA-1). (**B**) 15% SDS-PAGE under non-reducing conditions and Western blot analysis of fractions D1–E3 using anti-FIMA-1. (**C**) 1% AgGE of fractions D1–D9 and Western blot analysis using anti-FIMA-1; for comparison (**C**): fraction D7 from Figure 2 (S-500 before reduction).

As the Mr of FIM-A.1 in Complex II appeared differently in denaturing gels (SDS-PAGE; Figure 2C) and native gels (AgGE; Figure 2E), it might be possible that FIM-A.1 consists of oligomers under non-denaturing conditions. To test this possibility, we reduced and denatured FIM-A.1 of Complex II (as obtained from fractions D6 + D7 in Figure 2B) by boiling in β-mercaptoethanol and 0.1% SDS, and determined then the behavior of (monomeric) FIM-A.1 on an S-500 column again (Figure 3). 

The absorbance at 280 nm shifted drastically to lower Mr, but the PAS-positive mucin peak did not shift and was still detectable between fractions D2–D10 (Figure 3A). Furthermore, FIM-A.1 still peaked between fractions D3–D8 (Figure 3A,B). When analyzed by AgGE, the bulk of FIM-A.1 appeared at the same Mr as before the reduction process (Figure 3C). Thus, the reduction did not result in a significant shift of FIM-A.1 from Complex II to lower molecular mass.

### 2.2. Purification of X. laevis FIM-A.1 by SEC and Anion-Exchange Chromatography

**Figure 4:**
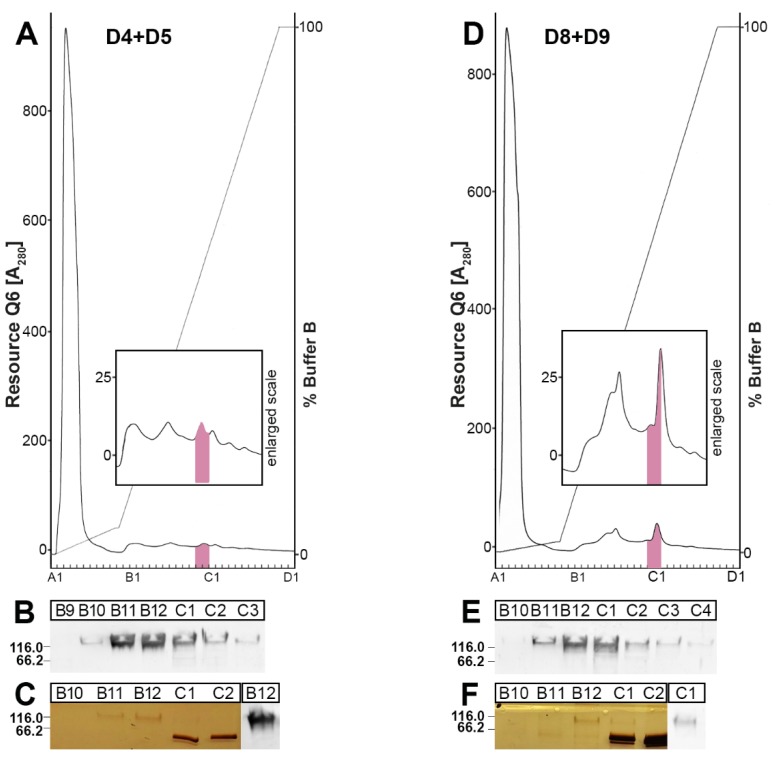
Purification of *X. laevis* FIM-A.1 by SEC and anion-exchange chromatography. (**A**) Fractions D4 + D5 (1 mL each) from the SEC of Figure 2 were separated by anion-exchange chromatography on Resource Q6. Elution profile as determined by absorbance at 280 nm (PAS-positive mucin fractions: pink). (**B**) 15% SDS-PAGE of fractions B9–C3 under reducing conditions and Western blot analysis using anti-FIMA-1. (**C**) 15% SDS-PAGE of fractions B9–C2 under non-reducing conditions and silver staining. For comparison, Western blot analysis of fraction C1 using anti-FIMA-1. (**D**) Separation of fractions D8 + D9 analogous as described in (**A**). (**E**) Western blot analysis of fractions B10–C4 using anti-FIMA-1. (**F**) Silver staining of fractions B10–C1 and Western blot analysis of fraction C1 using anti-FIMA-1.

In an attempt to purify FIM-A.1, the enriched fractions D4 + D5 and D8 + D9, respectively, from Complex II after SEC (Figure 2) were separated additionally by anion-exchange chromatography on a Resource Q6 column (Figure 4). PAS-positive mucin fractions were concentrated within just two fractions (B11/B12 and B12/C1, respectively; Figure 4A,D) and, within these fractions, FIM-A.1 also peaked (Figure 4B,E). Silver staining of these fractions showed that they contain highly enriched FIM-A.1 (Figure 4C,F).

### 2.3. In Vitro Binding Studies of Mucus Fractions with Radioactively Labeled FIM-A.1 (Overlay Assays)

**Figure 5:**
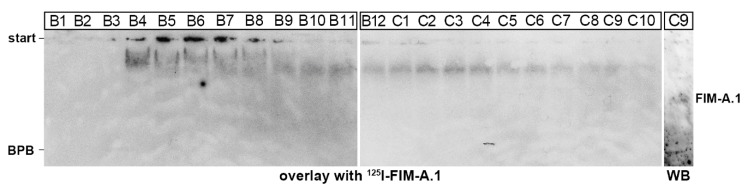
In vitro binding of ^125^I-labeled FIM-A.1 with mucin fractions from *X. laevis* skin (overlay assay). FIM-A.1 was purified from *X. laevis* skin via SEC (from Figure 2) followed by anion-exchange chromatography of fractions D4 + D5 (see Figure 4A). Then, fraction B11 containing purified FIM-A.1 (Figure 4A) was labeled with ^125^I. 1% AgGE of fractions B1–C10 after SEC from Figure 2 and hybridization of the blot with ^125^I-FIM-A.1 (autoradiography). The start and the dye bromophenol blue (BPB) are marked on the left. For comparison, FIM-A.1 was detected on the same blot by immunostaining with anti-FIMA-1 (WB, Western blot; C9).

As TFF domains show lectin activities, we tested here whether FIM-A.1 was able to bind mucins in Complex I and Complex II. Thus, FIM-A.1 was purified via SEC (Figure 2), followed by anion-exchange chromatography (Figure 4A) and radioactively labeled with ^125^I. Then, ^125^I-FIM-A.1 was used for in vitro binding studies (Figure 5) with the mucus fractions from Figure 1. FIM-A.1 was bound in Complex I (fractions B4–C10) by different high-molecular-mass proteins, probably mucins (Figure 5). There was binding with a very large entity at the start (B4–B8) as well as a smear underneath (B4–B8), and a third entity with a lower molecular mass (B7–C10). No binding was observed with proteins in Complex II and beyond (fractions C11–E8; data not shown).

## 3. Discussion

### 3.1. The Frog Integumentary Mucus Consists of Two Complexes with Different Molecular Masses

FIM-A.1 and FIM-C.1 are of different sizes (Figure 2C,F), and SEC using the S-500 column is capable of separating these mucins into Complexes I and II (Figure 2B). The predominant ≥ 116,000 band characteristic of FIM-A.1 represents the full-length sequence as this band is recognized by both the N-terminal (anti-FIMA-2) as well as the C-terminal antisera (anti-FIMA-1; Figure 2D). Interestingly, under reducing conditions, the C-terminal antiserum also recognizes a band below 14,000, which is present in all fractions positive for the typical ≥ 116,000 band (i.e., in Complex I and in Complex II) and is missing under non-reducing conditions (Figure 2C). It cannot be currently excluded that this band is the result of a partial processing of FIM-A.1 at a pair of basic amino acid residues, which would liberate a C-terminal peptide comprising of 50 amino acid residues [12].

Of special note, the high-molecular-mass Complex I does not only contain FIM-C.1, but also little FIM-A.1 (Figure 2B). However, the relative amount of FIM-A.1 in Complex I shows individual differences (data not shown). This observation is in agreement with a model, where little of FIM-A.1 is non-covalently bound to a high-molecular-mass mucin, such as FIM-C.1, and can be released by boiling in SDS, i.e., under non-reducing SDS-PAGE (Figure 2C). However, the bulk of FIM-A.1 does not interact with this mucin and appears separately in Complex II (Figure 2B). This model is supported by Figure 2E, where FIM-A.1 in Complex I (represented by fraction B6) shows a smear after AgGE towards higher molecular masses.

In contrast, FIM-A.1 in Complex II (represented by fractions D5 and D8; Figure 2E) appears with a molecular mass comparable with that of FCGBP (i.e., Mr > 650,000, [33]). Thus, the molecular mass of FIM-A.1 in Complex II after non-denaturing AgGE (>650,000; Figure 2E) appears to be much higher than under non-reducing denaturing SDS-PAGE (≥116,000; Figure 2C). One explanation would be that the extensive O-glycosylation of FIM-A.1 leads to a linear structure with an increased hydrodynamic radius when compared with coiled proteins resulting in an aberrant behavior after AgGE. Alternatively, the slower migration on AgGE could be a sign that FIM-A.1 in Complex II forms aggregates/oligomers under non-denaturing conditions.

To test the latter possibility, FIM-A.1 from Complex II (fractions D6 + D7, Figure 2) was reduced in boiling 4.7% β-mercaptoethanol/0.1% SDS and separated again on an S-500 column (Figure 3). As we could not observe a shift of FIM-A.1 towards lower molecular masses (Figure 3) compared with the original S-500 column (Figure 2), the oligomerization of FIM-A.1 is unlikely.

### 3.2. FIM-A.1 Binds to High-Molecular-Mass Mucins of Complex I

From Figure 2, it is clear that in Complex I, little FIM-A.1 is bound non-covalently to other mucin(s), e.g., FIM-C.1. To test possible lectin activities of FIM-A.1, this unusually short mucin was purified via SEC (Figure 2), followed by anion-exchange chromatography (Figure 4A), radioactively labeled and used for in vitro binding studies (Figure 5). Mainly binding to a large entity at the start and a smear underneath was observed, strongly indicating a binding to a high-molecular-mass mucin. Generally, binding mainly occurs in fractions B4–B8 positive for FIM-C.1 (compare with Figure 2B,F). Furthermore, the binding to a very high-molecular-mass protein/mucin not entering the gel would be an indication that FIM-C.1 might be the binding partner (compare with Figure 2F). In addition, binding to a somewhat smaller band was also observed (fractions B7–C10). This band clearly appeared with a higher Mr than FIM-A.1 and could also represent FIM-C.1, as this mucin is highly polymorphic due to alternative splicing [20].

Given the known lectin activities of TFF modules [29,30,31,32], one could hypothesize that FIM-A.1 binds as a lectin to the carbohydrate moiety of another mucin, such as FIM-C.1. Alternatively, another mucin, such as the TFF modules of FIM-C.1, might bind the carbohydrate moiety of FIM-A.1. This model is appealing as FIM-A.1 and FIM-C.1 are secretory products of different cells of the mucous glands, i.e., ordinary mucous cells and cone cells, respectively (Figure 1A) [13,26] and these cell types differ in their secretory granules as well as their lectin staining patterns [26]. The latter is indicative of different O-glycosylation of mucins from ordinary mucous cells and cone cells [26].

The occurrence of the two separate mucin Complexes I and II after SEC (Figure 2) could be explained by this model as well. FIM-A.1, as a secretory product of the numerous ordinary mucous cells, is the predominant integumentary mucin of *X. laevis*, whereas, e.g., FIM-B.1 and FIM-C.1 represent just a minor species. Thus, binding of FIM-A.1 to mucins of Complex I is limited by the amount of these mucins. This would also explain why we observed differences in the relative amount of FIM-A.1 bound to Complex I between different individuals (data not shown).

Currently, it is not clear what might be the physiological rationale of why frog skin mucins associate in two separate Complexes I and II and why they do not form one large complex. One explanation might be that frog skin mucus consists of different layers, e.g., a loose outer layer and an adherent inner layer, such as has been found in mammalian stomach and colon, respectively [34]. Thus, the unusually short FIM-A.1 might be a candidate for a potential loose outer layer.

Taken together, FIM-A.1 interacts in a non-covalent fashion (likely via lectin activities) with high-molecular-mass mucins of Complex I allowing the formation of a rather complex frog integumentary mucus network. Furthermore, there are even more TFF peptides and mosaic proteins containing TFF domains known in skin secretions of *X. laevis* as well as other amphibia, such as the granular gland products xP2/APEG [35] or “βγ-crystallin and trefoil factor” (βγ-CAT) [36], which could also contribute to this network. For example, there are first indications that xP2 might be associated with low-molecular-mass forms of the FIM-A.1 complex (fractions D7–E2 in Figure 1; data not shown). In addition, various galactose and lactose-binding lectins from *X. laevis* skin might be also involved [37,38].

## 4. Materials and Methods

### 4.1. Extraction of Proteins and Purification by SEC and Anion-Exchange Chromatography

Proteins were extracted from the skin (about 2.6 g) of *X. laevis* (purchased from the W. de Rover, Herpetological Institute, Turnhout, Belgium) with a 5-fold amount (*w*/*v*) of buffer (30 mM NaCl, 20 mM Tris-HCl pH 7.0) including protease inhibitors (0.5 mM benzamidin, 0.1 mM Pefabloc SC, 1 µg/mL leupeptin) in a Precellys^®^24 lyser/homogenizer analogous, as described previously [39].

Then, 8 mL of skin extracts were fractioned by SEC with the ÄKTA^TM^ FPLC system (Amersham Biosciences, Freiburg, Germany) as described (fraction numbering: A1-A12, B1-B12, and so forth) [40] using a HiPrep 16/60 Sephacryl S-500 High Resolution column (S-500; 20 mM Tris-HCl pH 7.0, 30 mM NaCl, including protease inhibitors, flow rate 0.5 mL/min, 2.0 mL fractions).

Additionally, anion-exchange chromatography was performed as reported previously [33,41] using a Resource Q6 column (Amersham Biosciences; salt gradient from 20 mM Tris-HCl pH 7.0 (buffer A) to 20 mM Tris-HCl pH 7.0 + 1 M NaCl (buffer B); flow rate 6.0 mL /min, 1.0 mL fractions).

### 4.2. SDS-PAGE, Agarose Gel Electrophoresis, and Western Blot Analysis

Non-denaturing AgGE (containing 0.1% SDS), denaturing SDS-PAGE under reducing or non-reducing conditions, and periodic acid-Schiff (PAS) staining for mucins (dot blot) were described previously [33,40,42]. Silver staining of proteins on polyacrylamide gels was according to an established protocol [33,43].

Western blot analysis after SDS-PAGE (electrophoretic transfer) or AgGE (capillary blot) was as reported [41]. All gels after non-reducing SDS-PAGE were subjected to post-in-gel reduction with 1% mercaptoethanol, as described previously [40]. Gels after AgGE were blotted onto nitrocellulose membranes, and for the detection with antisera, the proteins were additionally reduced on the membrane in situ with 1% mercaptoethanol at room temperature for 5 min.

Mucin FIM-A.1 was detected with the polyclonal antiserum anti-FIMA-1 (formerly: SPL-5; 1:1000 dilution) against the C-terminal synthetic peptide CFEKAVPVVNS as described previously [13]. Furthermore, the polyclonal antiserum anti-FIMA-2 (formerly: FIM-3; 1:1000 dilution; [44]) against the synthetic peptide CSVAPNMRVN, which is close to the predicted N-terminal of FIM-A.1, was used. Coupling to keyhole limpet hemocyanine was with *m*-maleimidobenzoyl-*N*-hydroxysuccinimide ester [44]. Production of the polyclonal antiserum anti-FIMC-1 (formerly: SKP-1; 1:1000 dilution) against the C-terminal of FIM-C.1 (synthetic peptide SVMNVPWCFYRT) was reported previously [20]. For comparison, FIM-B.1 was detected with the polyclonal antiserum anti-FIMB-1 (formerly: FIM-1; 1:1000 dilution) as described previously [22]. FCGBP was detected using a commercial polyclonal antiserum (PAP389Hu01, Cloud-Clone Corp., Katy, TX, USA) against amino acids 5176–5344 of human FCGBP. Bands were visualized with the enhanced chemiluminescence (ECL) detection system. The signal was recorded either with the GeneGnome system (Syngene, Cambridge, UK) or the Chemostar system (Intas Science Imaging Instruments GmbH, Göttingen, Germany) for each band within a given frame. The relative intensity of the signals was calculated (semi-quantitative analysis) using the GeneTools gel analysis software (Syngene, Cambridge, UK), setting the highest intensity in a series to 100%.

### 4.3. In Vitro Binding Studies with FIM-A.1 (Overlay Assays)

FIM-A.1 was purified via SEC on an S-500 column followed by anion-exchange chromatography on Resource Q 6 column of fractions D4 + D5. Then, 500 µL of fraction B11 of the Resource Q6 column was desalted via a PD-10 desalting column (GE Healthcare Europe GmbH, Freiburg, Germany), and the three fractions positive for FIM-A.1 were concentrated with a Concentrator plus (Eppendorf, Hamburg, Germany) to a volume of 15 µL each. Labeling of FIM-A.1 with ^125^I (iodogen method) and overlay assays with ^125^I-labeled FIM-A.1 were analogous, as described for TFF2 previously [41]. In brief, mucin containing fractions after SEC were separated by AgGE, blotted onto nitrocellulose membranes, hybridized with ^125^I-labeled FIM-A.1 (in 20 mM Tris-HCl pH 7.0, 2.5 mM CaCl_2_, 500 mM NaCl), and exposed to a film (autoradiography).

## Abbreviations

AgGE	Agarose gel electrophoresis
FIM	Frog integumentary mucin
PAS	Periodic acid-Schiff
SDS-PAGE	Sodium dodecyl sulfate-polyacrylamide electrophoresis
SEC	Size-exclusion chromatography
TFF	Trefoil factor family
